# A Computational Model of Oxytocin Modulation of Olfactory Recognition Memory

**DOI:** 10.1523/ENEURO.0201-19.2019

**Published:** 2019-08-28

**Authors:** Christiane Linster, Wolfgang Kelsch

**Affiliations:** 1 Computational Physiology Laboratory, Department of Neurobiology and Behavior, Cornell University, Ithaca, New York 14850; 2 RG Developmental Biology, Department of Psychiatry and Psychotherapy, Central Institute of Mental Health, University Heidelberg, Mannheim D-68159, Germany

**Keywords:** anterior olfactory nucleus, computation, olfaction, oxytocin, plasticity, social odors

## Abstract

Social recognition in mammals depends on complex interactions between sensory and other brain areas as well as modulatory inputs by specific neuropeptides such as oxytocin (OXT). Social recognition memory specifically has been shown to depend among others on olfactory processing, and can be probed using methods similar to those used when probing non-social odor memory. We here use a computational model of two interconnected olfactory networks in the mouse, the olfactory bulb (OB) and anterior olfactory nucleus, to propose a mechanism for olfactory short-term recognition memory and its modulation in social situations. Based on previous experiments, we propose one early locus for memory to be the OB. During social encounters in mice, pyramidal cells in the anterior olfactory nucleus, themselves driven by olfactory input, are rendered more excitable by OXT release, resulting in stronger feedback to OB local interneurons. This additional input to the OB creates stronger dynamics and improves signal-to-noise ratio of odor responses in the OB proper. As a consequence, mouse social olfactory memories are more strongly encoded and their duration is modulated.

## Significance Statement

Oxytocin has long been associated with modulating neural networks during social encounters. We here use a computational model to show how neural plasticity, modulation, and memory for social odors interact when animals encounter conspecific odors. To date the exact neural processes of oxytocin modulation of social odor memory are not elucidated; our modeling approach allows us to draw from a number of experimental data from different levels of analyses to create a coherent framework for how oxytocin modulates odor processing to match behavioral demands.

## Introduction

Social bonding and conspecific recognition in most mammals and rodents in particular relies on olfactory processing of conspecific odors. Specific neuropeptides such as oxytocin (OXT) and vasopressin as well as the neuromodulator dopamine have been shown to play critical roles for encoding and retrieval of conspecific odors (for review, see Walum and Young, 2018). The neural circuitry involved in these processes rely on olfactory information about conspecific odors processed first in the main olfactory bulb (OB) and relayed to brain areas involved in discrimination, social memory, bonding, and contextual information. The neuropeptide OXT has been proposed to shape these processes among other mechanisms by indirectly modulating signal-to-noise ratio (S/N) in the OB via activation of pyramidal cells in the anterior olfactory nucleus (Oettl et al., 2016; Oettl and Kelsch, 2018; Walum and Young, 2018). We here use a large-scale computation model to investigate the neural mechanisms and effects of enhanced S/N triggered by OXT on odor processing and memory. Our modeling results show how OXT modulation of inputs from the anterior olfactory nucleus (AON) to the OB could underlie behavioral effects on social odor recognition memory.

The first step in pair bond formation or other types of social memory is the formation of a precise neural representation of the conspecific’s odor, allowing for encoding and later recall. OXT has been proposed to play a crucial role in modulating olfactory representations in the OB (Walum and Young, 2018), indirectly via the AON. The OB is the first neural network processing odor information from sensory neurons. Its output neurons, mitral cells (MCs) project to a number of secondary olfactory cortices including the anterior olfactory nucleus. Olfactory representations are strongly modulated by local interneurons in the OB (Shipley et al., 1996), neuromodulators (Fletcher and Chen, 2010), as well as back projections from secondary olfactory cortices (Rothermel and Wachowiak, 2014). The AON expresses high levels of OXT receptors (Vaccari et al., 1998), receives projections from the paraventricular nucleus (Knobloch et al., 2012), and manipulations of OXT activity in the AON modulate social recognition memory in mice and rats (Oettl et al., 2016). AON projects heavily to the OB, with projections terminating on local interneurons and modulating OB spontaneous and odor-evoked activity (Boyd et al., 2012; Markopoulos et al., 2012; Rothermel and Wachowiak, 2014; Aqrabawi et al., 2016). Specifically, OXT modulates odor representations in the OB by lowering background activity and enhancing odor responses (Oettl et al., 2016; Oettl and Kelsch, 2018). One possible behavioral correlate of this modulation is increased social memory, as evidenced by increased duration of such memories when AON OXT receptors are artificially activated (Oettl et al., 2016; Oettl and Kelsch, 2018). We use a computational approach to combine several types of experimental knowledge from different labs to suggest how OXT inputs to AON could modulate olfactory recognition memory duration. Our model first shows that recognition memory can be implemented within OB bulb networks, as suggested by several pieces of evidence, and second that AON projection to the OB can modulate S/N in the OB, increasing olfactory detection and discrimination, as proposed in a recent review (Walum and Young, 2018). In addition to modulating S/N, plasticity processes in the OB are regulated by AON input. As a consequence, if AON neural activity and odor responses are changed by the influx of OXT in social recognition situations, AON modulation of OB processes is changed and results in increased plasticity. We propose that OXT release in the AON changes odor recognition memory and present behavioral results from mice supporting these predictions.

## Methods

### Network architecture

**Figure 1.**
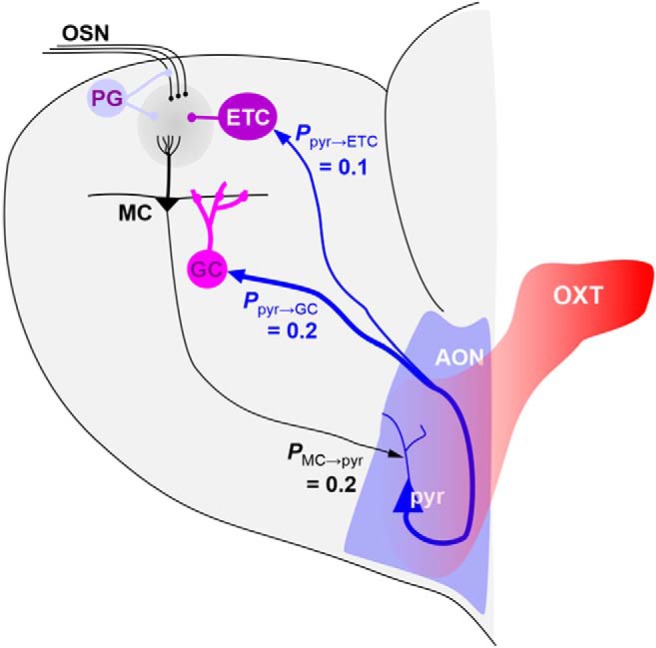
Model architecture. Simulated sensory neurons with broad responses to a chosen odorant make excitatory synapses onto PGs (inhibitory), ETs (excitatory), and MCs (output neurons) within a given glomerulus, conveying a common type of odor information to these neurons. MCs excite a large number of inhibitory GCs (via modifiable synapses) which in return convey inhibition only locally in one glomerular column. MCs project to 20% of AON Pyr cells, which excite each other through a dense network of association fibers (20% connectivity) and project back to OB interneurons in the glomerular (ETC; 10% connectivity) and GC layers (20% connectivity). OXT inputs modulate AON Pyr cell intrinsic properties.

The modeled OB network incorporates five neuron types: olfactory sensory neurons (OSNs), MCs, external tufted cells (ETs), periglomerular cells (PGs), and granule cells (GCs; Fig. 1). Each group is composed of 100 neurons organized in functional columns. MCs make synapses with 25% of GCs (*p*_MC-GC_ = 0.25) and GCs make inhibitory local synapses only (McIntyre and Cleland, 2016). Activity dependent plasticity to simulate olfactory habituation was implemented on synapses between MCs and GCs. The AON is represented by 100 pyramidal cells (Pyr). Because the connectivity between OB and AON is still poorly understood, synapses between mitral and Pyr cells were created randomly with each MC projecting to any Pyr cell with an equal probability of *p*_MC-Pyr_ = 0.2 as shown in Figure 1. Based on published data on AON projections to the OB, we included a relatively strong (*p*_pyr-GC_ = 0.2 with higher synaptic weights) connectivity from AON to OB GCs, as well as a weaker (*p*_P-ET_ = 0.1 with low synaptic weights) connectivity from AON to ET cells, which have been proposed to be the target of AON projections to the OB glomerular layer (Markopoulos et al., 2012). Intra-AON connections between Pyr cells were modeled with low synaptic weights and connectivity *p*_pyr-pyr_ = 0.25; AON interneurons were neglected at this time. As a consequence, AON inputs to the OB mainly increase GC layer inhibition in the OB, with a weaker increase in glomerular layer excitation (Fig. 1).

**Figure 2.**
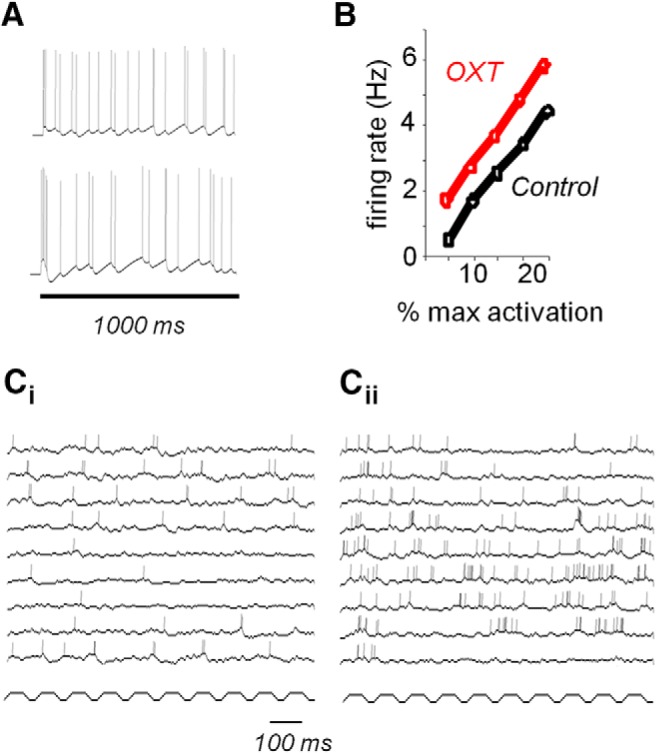
OXT modulation of AON Pyr cells. ***A***, AON Pyr cell responses to a 1 s current injection in the model under control (top) and OXT (bottom) conditions. (cf. Oettl et al., 2016, their Fig. S2). ***B***, Firing rates of AON Pyr cells as a function of activation in the model (cf. Oettl et al., 2016, their Fig. 4*B*). ***C***, Example AON cell activity under control (***C_i_***) and OXT (***C_ii_***) conditions in response to simulated odor inputs. Lower trace shows OSN potential fluctuations.

Modulation of AON Pyr cells by OXT was modeled by adjusting cell excitability (lowering firing threshold) to modulate the rheobase as shown by Oettl et al. (2016) and adjusting spike adaptation for these cells (through simulated Ca^+^-dependent K^+^ channels) to be less pronounced. Pyr cell parameters were adjusted to reflect experimental data under control and OXT modulation conditions (Fig. 2).

### Neurons and synapses

Our model is composed of single compartment leaky integrate-and-fire neurons, with the exception of MCs, which are modeled as two compartments. Changes in membrane voltage *v*(*t*) over time in each compartment are described by Equation 1:

(1) $$\tau \frac{dv\left(t\right)}{dt}+v\left(t\right)=V^{ext}\left(t\right),$$

where τ is the membrane time constant and *V* ^ext^(*t*) is the voltage change resulting from external inputs (synaptic or sensory).

Each one of the voltage changes due to external inputs *V* ^ext^ is a result of the synaptic strength of the connection from neuron *j* to neuron *i* (*w_ij_*) and the respective synaptic conductance in cell *i* at time *t* [*g_i_*(*t*)]. *E_N,ij_* is the Nernst potential of the synaptic current and *v_i_*(*t*) is the membrane potential of the postsynaptic neuron *i*, as described in Equation 2:

(2) $$V_{i}^{ext}\left(t\right)=w_{ij}g_{i}\left(t\right)\left[E_{Nij}-v_{i}\left(t\right)\right].$$

The communication between neurons happens via discrete spikes. The spiking output *F*(*v)* of a given neuron *i* is a function of its membrane potential *v* and the minimal threshold and saturation threshold of the output function, *θ* ^min^ and *θ* ^max^. Where *F_i_*(*v*) = 0 if *v* ≤ *θ* ^min^ and *F_i_*(*v*) = 1 if *v* ≥ *θ* ^max^ and *Fi*(*v*) increase linearly between θ_min_ and θ_max_.

*F*_i_(*v*) defines their instantaneous firing probability and OXT modulation decreases θ_max_ to increase excitability. The time course of the conductance change is calculated as follows:

(3) $$g_{i}\left(t\right)=g^{\max}\left(e^{-\frac{t}{\tau_{1}}}-e^{-\frac{t}{\tau_{2}}}\right),$$

where *g* ^max^ is a constant with no unit representing the maximum conductance of a given channel and is equal to 1 (synaptic strength is scaled by the synaptic weight *w*), whereas τ_1_ and τ_2_ are the rising and falling times of this conductance. After firing, the spike of each spiking-neuron is reset to *V*_rest_.

Spike adaptation was implemented in Pyr cells as a calcium-dependent K^+^ channel, which leads to a hyperpolarizing current in the cell. The calcium variable was dependent on the neuron’s firing and increased with each spike and slowly decreased over time using a first order differential equation with τ_ca_ as time constant (for detailed parameters, see Table 1) and *V*_N_ = −90 mV and *A* ^ahc^ the amplitude of the effect for the K^+^ conductance. OXT modulation affected the maximal conductance of the calcium-dependent K^+^ current into the cell.

**Table 1.** Computational modeling parameters

OSN	τ = 1 ms; *V*_rest_ = −65 mV; θ^min^ = −65 mV; θ^max^ = −55 mV
Mitral	τ = 5 ms; *V*_rest_ = −65 mV; θ^min^ = −64 mV; θ^max^ = −55 mV
PG	τ = 2 ms; *V*_rest_ = −65 mV; θ^min^ = −65 mV; θ^max^ = −60 mV
GC	τ = 4 ms; *V*_rest_ = −65 mV ; θ^min^ = −64 mV; θ^max^ = −60 mV
ET	τ = 2 ms; *V*_rest_ = −65 mV ; θ^min^ = −65 mV; θ^max^ = −60 mV
Pyr	τ = 10 ms; *V*_rest_ = −65 mV; θ^min^ = −62 mV; θ^max^ = −55 mV/−60 mV^*^;
OSN to PG	w= 0.0015; *E*_N_ = +70 mV; τ_1_ = 1 ms; τ_2_ = 2 ms
OSN to Mi (apical)	w = 0.015; *E*_N_ = +70 mV; τ_1_ = 1 ms; τ_2_ = 2 ms
OSN to ET(apical)	w = 0.0015; *E*_N_ = +70 mV; τ_1_ = 1 ms; τ_2_ = 2 ms
PG to Mi (apical)	w= 0.002; *E*_N_ = −5 mV; τ_1_ = 2 ms; τ_2_ = 4 ms
ET to Mi (apical)	w= 0.0015; *E*_N_ = 70 mV; τ_1_ = 1 ms; τ_2_ = 2 ms
Mi (soma) to GC	w_naive_= 0.0001 *E*_N_ = +70 mV; τ_1_ = 1 ms; τ_2_ = 2 ms ; *p* = 0.25 ; α = 0.001 ; τ_forget_ = 12.5 min.
GC to Mi (soma)	w= 0.0015; *E*_N_ = −10 mV; τ_1_ = 2 ms; τ_2_ = 4 ms ; local only
Mi (soma) to Pyr	w= 0.007; *E*_N_ = +70 mV; τ_1_ = 1 ms; τ_2_ = 2 ms ; *p* = 0.20
Pyr to ET	w= 0.0015; *E*_N_ = +70 mV; τ_1_ = 1 ms; τ_2_ = 2 ms ; *p* = 0.1
Pyr to GC	w = 0.015; *E*_N_ = +70 mV; τ_1_ = 1 ms; τ_2_ = 2 ms ; *p* = 0.2
Pyr adaptation	A^ahc^ = 10|0.2^*^; *E*_N_ = −90 mV; τ^ahc^ = 100 ms

τ, Membrane time constant; *V*_rest_, resting membrane potential; θ^min^, spiking threshold; : θ^max^, saturation threshold; w, synaptic weight; *E*_N_, reversal potential; τ_1_, rise time; τ_2_, decay time; A^ahc^, after-hyperpolarization magnitude; τ^ahc^, calcium accumulation time constant.

* Different values are without/with OXT modulation, respectively.

In the simulations presented here, simulated exposure to an odorant induced activity-dependent plasticity of synapses from MCs to GCs. Synaptic strengths were first calculated from the parameters given in Table 1. During simulated odor exposures, synapses between MCs and GCs underwent synaptic potentiation:

(4) $$w_{ij-new}=w_{ij-old}+\alpha *\sum_{t1}^{t2}x_{i}\left(t\right)*\sum_{t1}^{t2}x_{j}\left(t\right),$$

where *w_ij_* is the synaptic strength between the presynaptic MCs and postsynaptic GCs α is the rate of potentiation and *x_j_* and *x_i_* are the total numbers of spikes emitted by the presynaptic and postsynaptic cells during the preceding sniff cycle between *t*1 and *t*2. During the delay between encoding and recall, synaptic decay exponentially with a long time constant (12.5 min) to simulate forgetting. This time constant was adjusted to match experimental control data with mice undergoing an object recognition task with non-social odors.

### Implementation

**Figure 3.**
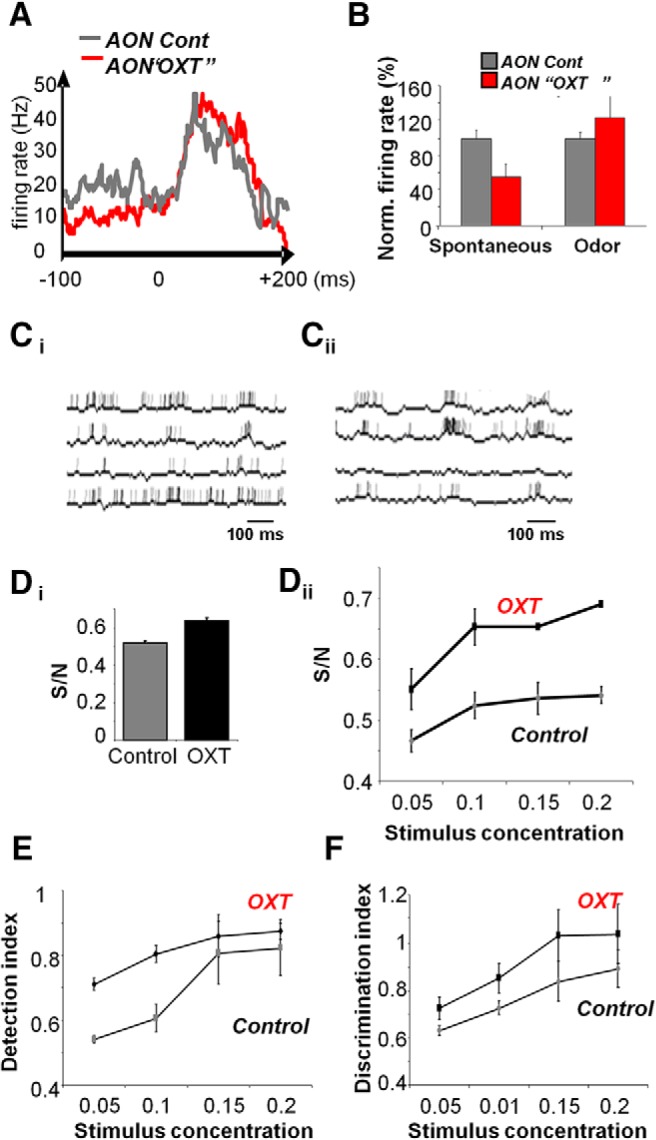
Modulation of OB odor responses by AON. ***A***, MC firing rate during a single respiration cycle in response to odor stimulation under control and OXT (cf. Oettl et al., 2016, their Fig. 7*B*). ***B***, MC firing rates normalized with respect to control (no OXT) conditions during spontaneous activity and odor-evoked activity (cf. Oettl et al., 2016, their Fig. 7). ***C***, Example MC traces during respiration-modulated odor responses under control (No OXT; ***C_i_***) and OXT in the AON (***C_ii_***) conditions. Note the decrease of spontaneous activity and increase in odor responsiveness. ***D***, OB S/N modulation by AON OXT modulation. ***D_i_***, The average (+/− standard error) S/N for control and OXT simulations. ***D_ii_***, The average (+/− standard error) S/N for OXT and control simulations as a function of stimulus concentration (ratio of maximum odor concentration in the model). ***E***, The graph shows the detection index (average +/− standard error) for odorants varying between 0.05 and 0.2 of the maximal stimulus concentration in the model. ***F***, The graph shows the discrimination index (average +/− standard error) between two simulated odorants as a function of stimulus concentration.

**Figure 4.**
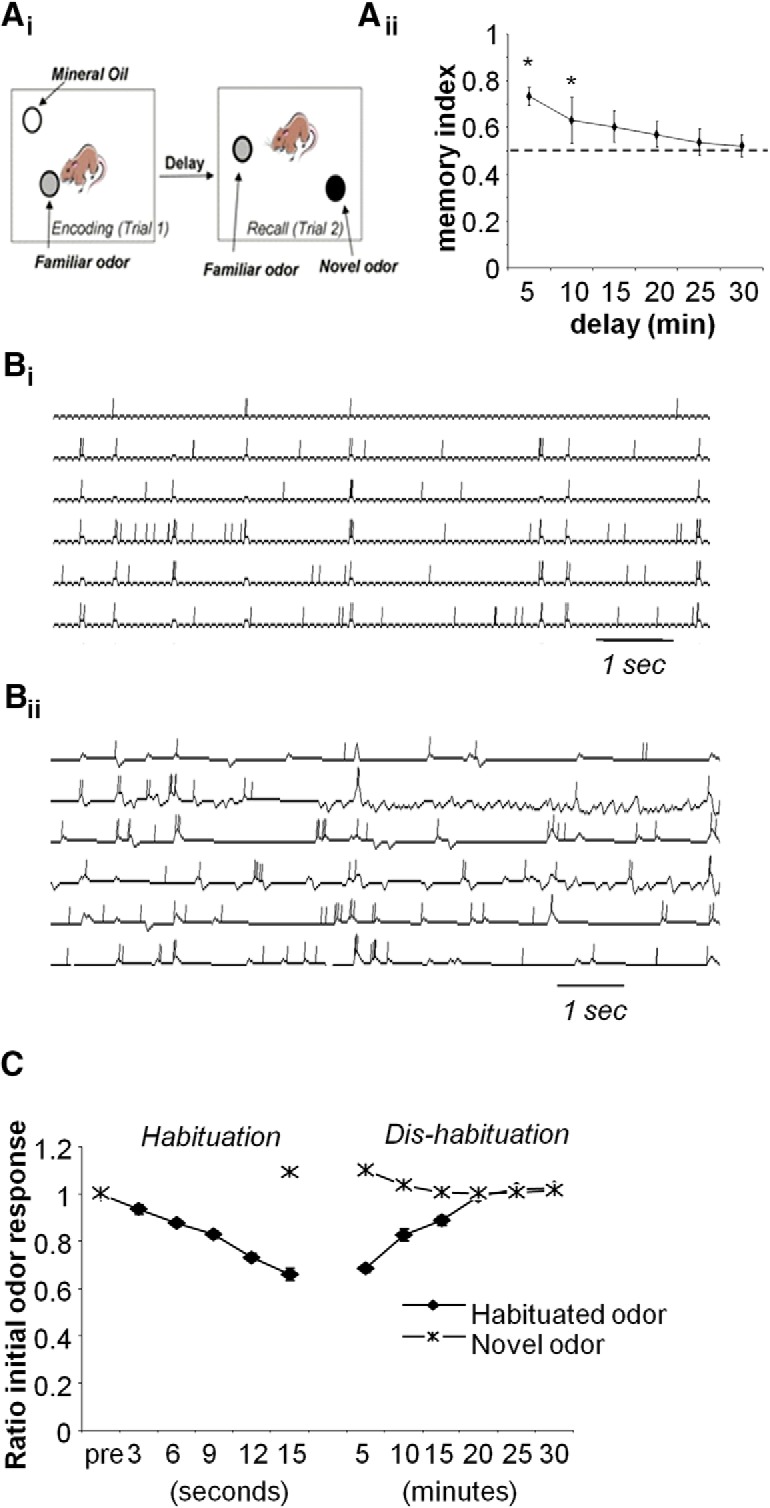
Odor object recognition in mice and in the computational model. ***A_i_***, Schematic representation of behavioral testing in mice. Mice are tested on a 60 ×60 cm platform on which odor stimuli are presented in Eppendorf tubes positioned in custom-made holders. Mice are left to investigate the odor for a period of 2 min. After a variable delay (5, 10, 15, 20, 25, or 30 min) mice are reintroduced on the platform with the familiar and a novel odor and investigation time in response to both is recorded. ***A_ii_***, Memory index (fraction of spend time investigating the novel odor) is shown as a function of the delay (average +/− standard error). Mice investigate the novel odor significantly more than the familiar odor after 5 and 10 min delays as indicated by asterisk. The graph shows average memory index ± SE. ***B_i_***, Example receptor neuron traces in response to short bouts of “investigation” in the model. ***B_ii_***, MC responses during the encoding trial when MC to GC synapses increase in an activity-dependent manner. Note that MC responses to the investigation bouts decrease over time. ***C***, The evolution of average MC odor responses in the model during encoding (3–15 s) and during the delay (5–30 min) when no odors are presented.

**Figure 5.**
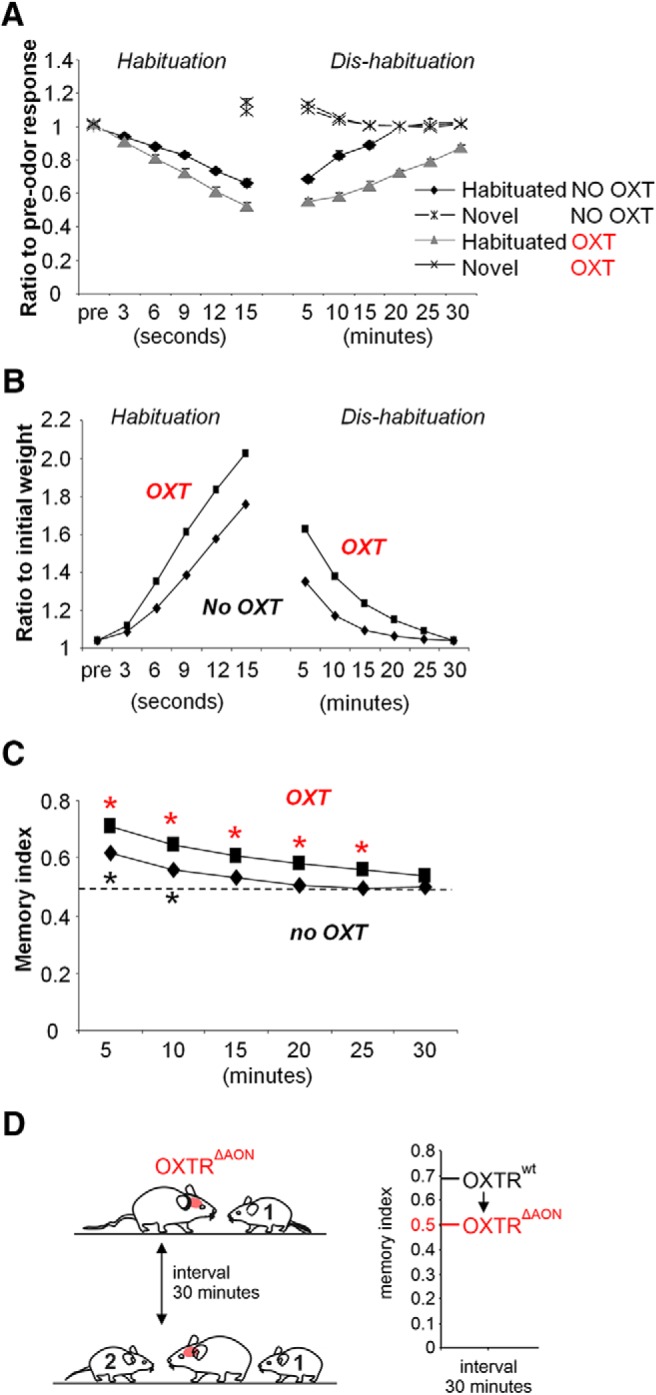
OXT modulation of AON increases plasticity and memory duration in the OB. ***A***, Modulation of AON by OXT increases habituation in the OB. The graph shows average MC responses to odor stimulation in response to the habituated odor or a novel odor with (OXT) or without (NO OXT) modulation in the AON during encoding. During recovery (dis-habituation) MCs modulated in the presence of OXT recover to a lesser degree (note there is NO OXT during recovery). ***B***, The graph shows changes in synaptic weights during habituation and dis-habituation phases with OXT during habituation (OXT) or control conditions (No OXT). ***C***, The resulting memory index (responses to novel odor/sum of responses) shows that when OXT is simulated in the AON, model MCs respond significantly more to a novel than to the habituated odor for longer delays (up to 25 min) when OXT was present during the encoding phase. ***D***, Summarizing scheme of the impaired loss of recognition memory without OXT receptor recruitment in the AON (left) and comparison of memory index in control (OXTR^wt^) mice and mice with OXT receptors deleted in the AON (OXTR^DAON^; from Oettl et al., 2016). Mice with OXTRs intact remembered the conspecific after a delay of 30 min, whereas mice with deleted OXTRs did not (similar to mice encoding non-social odors; Fig. 2*A*). Asterisk indicates a significant difference between responses to familiar and novel odors at that delay.

All simulations were implemented using the C programming language in a Linux environment (Ubuntu 14.04 LTS x64) on an Intel desktop computer, with Euler integration method for the differential equations with a time step of 1 ms for short-term (1 s) simulations (Figs. 2, 3) and 10 ms for long-term habituation-dishabituation simulations (Figs. 4, 5).

### Code accessibility

The code/software described in the paper is freely available online at http://modeldb.yale.edu/257940. The code is available as Extended Data 1.

10.1523/ENEURO.0201-19.2019.ed1 Extended data 1 Code used to perform the simulations presented here. Download Extended Data 1, ZIP file.

### Behavioral methods

Ten C57-black6 mice (male and female) between 3 and 6 months old at the beginning of the experiment group housed with *ad libitum* access to food and water. Experiments were run in a 60 × 60 cm platform on which two custom made holders for odors were placed. On each testing day, a mouse was first placed on the platform in the presence of an Eppendorf vial containing mineral only and one containing an odor diluted to ∼1 Pa vapor partial pressure in mineral oil. The mouse was left to investigate the two vials for a 2 min encoding period and then placed back into its home cage. After a variable delay of 2, 5, 10, 15, or 20 min, the mouse was put back onto the platform in the presence of the familiar and a novel odor for a recall period of 2 min. Each mouse was tested once a day, every other day for 6 testing days total (1 d per delay). On each testing day and for each mouse, a different pair of odors was assigned as familiar and novel in a pseudorandom and counter balanced manner such that each mouse was tested with a pair only once (Table 2). The location of the odors was varied in a pseudorandom fashion on each trial. Mice were videotaped and behavior was scored online. For each trial and odor, we scored for how long mice investigated the odor by measuring the time spent actively investigating in a 1 cm radius around the odor source. Data were analyzed by comparing the investigation times in response to novel and familiar odor during the recall trial. A significant difference in these indicated that a mouse remembered the encoded odor. Data are represented as memory index (*R*_novel_/(*R*_novel_+*R*_familiar_) in the graphs. The experimental design of this study was based on a previous study in rats testing the parameters of olfactory recognition memory including memory duration, numbers of odors remembered as a function of time and memory specificity for odors as a function of time (Hackett et al., 2015). We have used the same paradigm in rats to investigate the effects of stress on rat odor memory performance (Manella et al., 2013, 2016). We chose this paradigm because the basic setup is similar to that commonly used in social recognition and memory studies (Oettl and Kelsch, 2018).

**Table 2.** Odors and dilutions (μl per 50 ml) used for the behavioral experiments

Odor pair	Odor	Dilution (μl in 50 ml)	Odor	Dilution (μl in 50 ml)
1	Butanol	16	Butyl propionate	10.9
2	Geraniol	1250	(−)Carvone	2360
3	Butanal	10	Octanal	74
4	3-heptanone	323	Methyl sacilate	1740
5	Octanoic acid	6870	Methylvalerate	30
6	1-heptanone	29	(+)limonene	102

All animal procedures were performed in accordance with Cornell University’s animal care committee's regulations.

## Results

In this section, we first show the implementation of OXT modulation on AON Pyr cells, as well as indirect effects on OB odor responses (Fig. 2). Next we tested to what extend the experimentally observed S/N modulation affects OB odor detection, discrimination and synchronization properties in the model (Fig. 3). We then show how plasticity within the OB can account for experimental results on odor recognition (Fig. 4) and to what extend OXT modulation increases plasticity in the OB (Fig. 5). Overall our simulations propose a mechanism by which OXT activation of AON supports known behavioral observations and predict modulatory effects on odor detection thresholds and discrimination, as suggested recently by Walum and Young (2018).

### Activation of AON Pyr cells by OXT

Effects of OXT on AON Pyr cells were simulated by adjusting spike thresholds and spike rate adaptation as described experimentally (Oettl et al., 2016) and are illustrated in Figure 2. OXT increases AON Pyr cell responses to current injections as well as spontaneous activity. These effects were modeled to match those published in Oettl et al. (2016). Figure 2*A* shows simulation of two model AON Pyr cells responding to a 1 s continuous simulated current injection under control (no OXT) conditions. The effect of OXT modulation on AON neurons to brief stimuli is illustrated in Figure 2*B*; the graph shows the effect of OXT modulation by comparing average firing rates of AON neurons under control and OXT conditions when stimulated with a brief (1 s) current input. Figure 2*C* shows example AON cells under control (*C_i_*) and OXT (*C_ii_*) conditions in the complete model, as well as the simulated OSN potential (last row in each graph). Note that AON cells fire more rapidly due to increased excitability and decreased spike rate. AON neurons are driven by input from OB MCs.

### Increased excitability of AON neurons by OXT change odor responses and S/N in the OB

We then tested how AON activation by OXT affects odor responses in the OB. When AON Pyr cells have increased excitability under OXT modulation, their impact on OB neural activity is more pronounced. Experimentally as well as in our model, the result is a higher S/N in response to odorants. Figure 3*A* shows average firing rates of odor responsive MCs in the OB model over a single respiratory cycle in control and OXT simulations. Note the decrease of spontaneous activity (due to AON activation of inhibitory interneurons) and the increase in peak odor response (due to AON activation of excitatory interneurons). Figure 3*B* quantifies the changes in S/N by graphing average MC firing rates during spontaneous and odor-evoked activity. Figure 3*C* shows OB MC responses to a randomly chosen odor stimulation under control (*C_i_*) and OXT (*C_ii_*) simulations. Note the decrease of spontaneous activity, increase in odor responses and increased dynamics in the OB traces. We then quantified S/N in the model by calculating the average spontaneous (*R*_S_) and odor-evoked (*R*_O_) activities in MCs and measuring S/N = *R*_O_/(*R*_O_+*R*_S_) (Hasselmo et al., 1997; Escanilla et al., 2010). 20 instances of the model were run without and with randomly chosen odorant stimulations at a specific odor concentration and the results are depicted in Figure 3*D*. In the model, S/N in the OB is significantly improved for a given range of odor concentrations by OXT modulation of the AON (ANOVA comparing average S/N for Control and OXT simulation: *F*_(1,22)_ = 28.893; *p* < 0.001 in Fig. 3*Di*, with significant differences between groups at the two higher concentrations; *p* < 0.0125 with corrections for multiple comparisons) and S/N correlated with odor concentration under both conditions (*r* = 0.643, *p* < 0.05 for control and *r* = 0.763, *p* < 0.01 for OXT; Fig. 3*Dii*).

### Increased detection and odor discrimination in the OB under AON - OXT modulation

Next we tested to what extend OXT modulation of AON effects odor representations at the output of the OB by calculating odor detection and discrimination thresholds. These output representations drive cortical processing and define to a certain degree the quality of olfactory memories stored (Devore et al., 2014; de Almeida et al., 2016). To measure odor detection, we compared population MC responses (average spike rates) during spontaneous activity to that during odor responses of decreasing odor concentrations. Twenty different instances of the model were run either with no odor stimulation or stimulation with a randomly chosen odor at four very low levels of odor stimulation (0.05, 0.1, 0.15, and 0.2 of the maximally possible stimulation level). Odor detection in the OB, i.e., the difference between MC odor responses and MC spontaneous activity (depicted as 1-correlation between the representations), was significantly improved by AON modulation as well as by odor concentration [effect of group (OXT, control): *F*_(1,22)_ = 4.787; *p* = 0.04) and correlated with odor concentration in both cases (*R* = 0.727 for OXT and *R* = 0.746 for control, *p* < 0.01 in both cases]. In particular, detection indices between groups were significantly different between conditions at the two lowest concentrations (*p* < 0.0125 with corrections for multiple comparisons; Fig. 3*E*). Odor detection, measured as a significant difference between spontaneous and odor-evoked responses was significant at the two highest concentrations for control simulations and for the three highest concentrations for OXT stimulations, suggesting that OXT modulation allows the model to detect the presence of odorants at very low odor concentrations.

Discrimination between pairs of odorants was assessed by comparing population MC responses (average spike rates) at the output of the OB in response to two different randomly chosen stimuli of varying concentrations (Fig. 3*F*). Similar to detection, discrimination improved with increasing odor concentration (*r* = 0.756, *p* < 0.01 for control and *r* = 0.766, *p* < 0.01 for OXT). There is a significant overall difference in discrimination indices (depicted as 1-correlation) between the two simulations (*F*_(1,22)_ = 4.866, *p* = 0.038) with significant differences at the two lowest odor concentrations (*p* < 0.0125 with corrections for multiple comparisons).

### Increased excitability of AON neurons by OXT modulates OB plasticity and odor object recognition memory

Experimental data strongly points to the OB as the location for olfactory recognition memory formation at the time scales used in social and odor recognition paradigms (for review, see Wilson and Linster, 2008). First, the duration and specificity of olfactory recognition memory can be modulated via manipulations local to the OB (Mandairon and Linster, 2009; Escanilla et al., 2010; Dillon et al., 2013; Manella et al., 2013). Second, NMDA receptors in the OB are implicated in the formation of this type of memory (McNamara et al., 2008; Chaudhury et al., 2010) and OB MC odor responses adapt in response to repeated odor stimulation in a manner similar to behavioral habituation (Chaudhury et al., 2010). Third, broad lesions of centrifugal inputs to the OB do not impair habituation memory formation (Kiselycznyk et al., 2006). We used our OB model to test to what extend local changes in synaptic strength could lead to habituation memory formation and social recognition and to what extend this phenomenon can be modulated by OXT in the AON.

We first modeled olfactory recognition to non-social odors memory in mice. These tests would correspond to a no-OXT control situation since can assume OXT release to be triggered by social encounters and odors. In experimental tests, mice were first presented with an odor and a blank (often the carrier, mineral oil) and allowed to investigate these two stimuli. After the encoding trial and a variable delay, mice were presented with the familiar odor and a novel odor and investigation times were scored offline. Odor recognition memory is present if the familiar odor is significantly less investigated during the recall trial than the encoding trial, and discrimination can be assessed as the difference or ratio in novel and familiar odor investigation (Manella et al., 2013; Hackett et al., 2015). Figure 4*A* shows a schematic of such an experimental setup and behavioral results obtained in mice. In this experiment, mice remembered the encoded odor for a maximum of 10 min after an encoding period of 2 min. ANOVA with familiar and novel odor as within-subjects factor and delay as between-subjects factor showed a significant effect of odor (*F*_(1,54)_ = 18.299, *p* < 0.001) as well as a significant interaction between odor and delay (*F*_(6,54)_ = 2.724; *p* = 0.029). *Post hoc* comparisons showed a significant difference in investigation of familiar and control odor after delays of 5 min (*p* = 0.001) and 10 min (*p* < 0.008) minutes, but no longer delays. The graph in Figure 4*Ai* shows the memory index (*R*_novel_/(*R*_novel_+*R*_fam_) as a function of delay.

In the model, investigation bouts during encoding were simulated as an average of 15 randomly presented odor stimulations lasting an average of 200 ms over the course of a 15 s encoding window (Fig. 4*Bi* shows example OSN; and Fig. B*ii* shows example MC responses to investigation bouts). MCs depicted respond to odor stimulation with a mix of excitation and inhibition; the number of action potentials emitted decreases over time. At the MC to GC synapse, activity-dependent plasticity (see Materials and Methods) was implemented in such a manner that odor responsive GCs would receive stronger MC inputs from odor responsive MCs and as a consequence MC odor responses decrease with successive investigation. The obtained decreases of MC odor responsiveness correspond to what was observed experimentally in rat MCs (Chaudhury et al., 2010), and its behavioral correlate is a decrease in odor investigation over time. The graph in Figure 4*C* shows the evolution of average MC firing rates during odor stimulation over time of habituation (0–15 s) and dis-habituation (5–30 min) for the habituated and a novel odor under control conditions (no OXT). MC average firing rates decreased significantly with time (R = −0.960 with *p* < 0.01 using Pearson’s *R*) and later increased significantly during recovery time (R = 0.965 with p < 0.01 using Pearson’s *R*). Odor responses slowly recover: when tested after 5 or 10 min: odor responses are below baseline (overall effect of delay *F*_(6,63)_ = 67.4; *p* > 0.01 and *p* < 0.01 in pairwise comparisons between baseline activity in response to the familiar odor and activity after 5 and 10 simulated recovery minutes) but have recovered to 100% of baseline after 15 simulated minutes (Fig. 4*C*).

We then tested how increased excitability of AON neurons by OXT modulates memory formation in the OB model. To this purpose we ran the same simulations with and without OXT modulation of AON model neurons and then computed the memory index for control and OXT simulations to test to what extend AON OXT would extend memory duration beyond that obtained in control simulations with no other parameters changed.

Rendering AON Pyr cells more excitable by OXT slightly increased habituation learning in the OB model. MCs habituated to a somewhat greater degree over the same time course (Fig. 5*A*) and as a consequence dis-habituated less fully over the course of 30 simulated minutes (Fig. 5*A*). Note that OXT modulation was only simulated during encoding, not recall. This was due to more rapid changes in synaptic weights between MCs and GCs (Fig. 5*B*) because of additional AON activation of OB GCs and (indirectly) MCs. The memory index in simulations with OXT is increased to longer delay times as depicted in Figure 5*C*; in the model, responses to the familiar odor are now significantly higher than baseline up to an interval of 25 min (significant effect of delay (*F*_(6,63)_ = 189.26; *p* < 0.001 and *p* < 0.01 in pairwise comparisons (LSD) for delays of 5. 10, 15, 20, and 25 min) compared with 10 min when OXT modulation is not simulated (see results in previous paragraph). Figure 5*D* shows a summary of corresponding behavioral results published by Oettl et al. (2016) in which genetically deleted OXT receptors in the AON decreased odor memory duration: whereas control mice remembered a conspecific after an interval of 30 min, OXT receptor-deficient mice in the AON did not. This corresponds to a shorter recovery time in the model when OXT is not included during encoding (no OXT). Our simulations suggest that in social recognition situations, OXT release activates AON Pyr cells, which in turn drive OB cells resulting in faster synaptic plasticity during exploration and longer memory.

## Discussion

Modulation of neural networks by OXT and other modulators changes neural computations and can result in a number of seemingly unrelated behavioral outcomes. Using a large scale computational model of the olfactory system we here show how experimentally proposed regulation of S/N (Oettl and Kelsch, 2018; Walum and Young, 2018) can explain results on social memory duration. We first show how OXT modulation of anterior olfactory nucleus can change bulbar MC odor responses by increasing activity of projections to local interneurons (Markopoulos et al., 2012; Rothermel and Wachowiak, 2014). Odor responses in the OB are rendered more robust and less noisy when AON Pyr cells, themselves driven by bulbar input, are set to a state of higher excitability and show less spike rate adaptation in response to OXT influx (Oettl and Kelsch, 2018). Interestingly, the observed changes in bulbar processing lead to enhanced odor detection and discrimination in the model, reminiscent of results pertaining to noradrenergic modulation of bulbar processing (Escanilla et al., 2010, 2012; Linster and Escanilla, 2019). Most importantly our modeling results show that these same neural processes can underlie the changes in social recognition memory measured experimentally: increased bulbar S/N via OXT modulation of the AON increases odor learning in the OB in a manner that leads to a longer lasting memory (Oettl and Kelsch, 2018, their Fig. 6). These modeling results put cellular, network and behavioral results in perceptive with each other and explain the neural mechanisms by which OXT can modulate social recognition memory.

The OXT system, implicated in social behaviors (Walum and Young, 2018), has effects over a wide array of neural networks including olfactory areas. It has been specifically implicated in olfactory processing, an important modality of social interactions in rodents. We have recently shown a strong effect of OXT modulation on the duration of social recognition memory (Oettl et al., 2016), in conjunction with modulation of olfactory neural networks. Our model explores both where olfactory short-term recognition memory may be implemented and what the mechanisms of OXT modulation could be. Based on ample previous data from our laboratory, we implemented neural mechanisms for recognition memory in the OB network itself. The mechanism we propose, short-term plasticity on MC to GC synapses, are supported by a number of experimental results from our lab and others. First, local manipulation of bulbar networks can modulate recognition memory (Mandairon and Linster, 2009; Escanilla et al., 2010; Dillon et al., 2013; Manella et al., 2013). Second, the formation of habituation memory has been shown to be dependent on NMDA receptor function in the OB in both mice and rats (McNamara et al., 2008; Chaudhury et al., 2010), and OB MC habituate to odorants over the same time scales observed behaviorally and this habituation is NMDA receptor dependent (Chaudhury et al., 2010). Third, lesions of feedback projections to the OB did not impair the formation or recall of these types of memories (Kiselycznyk et al., 2006). Thus, all evidence points to a local bulbar mechanism of memory formation, modulated by extrinsic projections and behavioral state, including projections from the AON. Decrease of AON function did not impair mice’ ability to habituate or dis-habituate to odors (Aqrabawi et al., 2016), suggesting that AON inputs are not needed for memory formation per se, but rather modulate this function depending on behavioral state. We propose that over a short time of odor presentations, MC responses to this specific odor decrease because of increased local inhibition; this effect returns to baseline with a relatively short time constant of 10–15 min, as evidenced by behavioral testing and electrophysiological recordings (Chaudhury et al., 2010). We adjusted these parameters to match data obtained from non-social odor recognition experiments (Fig. 4). When AON input to the OB is modulated by OXT during a simulated social encounter, OB responses are less noisy, S/N increases in the OB and as a consequence, changes in synaptic transmission in the OB are stronger, creating a longer lasting odor memory. Thus, although the site of memory formation is proposed to be the OB, AON processes modulate plasticity and increase memory duration as observed in behavioral experiments using social encounters rather than non-social odors. Interestingly, when OXT receptors in the AON are nonfunctional, social recognition memory decreases in duration to match that obtained with non-social odors, supporting the idea that AON inputs modulate, rather than directly create, this memory.

In summary, we here propose that S/N in the OB and short-term memory duration are directly related. These ideas are supported by a wealth of data, including for example data showing that this type of olfactory memory is modulated in duration by noradrenergic projections to the OB (Devore and Linster, 2012; Linster and Escanilla, 2019). The OB integrates sensory and higher-order inputs; as a consequence, it is ideally situated to create sensory representations that reflect the current behavioral state. Modulation of OB processing directly affects memory formation and habituation memories at this time scale have been located to the OB proper. Olfactory processing in a state-dependent manner is not a simple feedforward system, but rather is distributed in a number of interconnected networks that can affect each other. State-dependent information, such as the presence of a con specific, a predator, a stressor, or a reward can modulate processing at many different levels including the first olfactory network, the OB. Our model shows that social interactions exploit the same olfactory networks underlying non-social olfactory processing and modulate their function to adjust to current behavioral demands.

## Synthesis

Reviewing Editor: Tatyana Sharpee, The Salk Institute for Biological Studies Decisions are customarily a result of the Reviewing Editor and the peer reviewers coming together and discussing their recommendations until a consensus is reached. When revisions are invited, a fact-based synthesis statement explaining their decision and outlining what is needed to prepare a revision will be listed below. The following reviewer(s) agreed to reveal their identity: Daniel Wesson.

In this paper the authors use previously published data to build models which support the conclusion that oxytocinergic changes in olfactory bulb are related with short term memory, which they discuss holds importance in considering the influence of oxytocin for social memory. There are several novel and/or timely results, including how the anterior olfactory nucleus influences the olfactory bulb and the influence of oxytocin on the olfactory system.

Reviewer 1 minor comments:

The authors seem to propose that their modeling uncovers how “non-social olfactory processing” may underlie social interactions (e.g., towards the end of the discussion). I'm not sure I agree with this conclusion, but am open to the authors rebutting. My logic is that the bulb has long been known a critical mediator of social behavior, with bulbectomy and other manipulations influencing a wide array of rodent social behaviors. So it doesn't seem so simple as to suggest that the bulb is a non-social odor processing network. Perhaps I'm reading more into this than the authors intend. But it does read literally that the authors are positing that the bulb is for non-social olfactory processing, which is not true. How else would social odors enter the brain?

Several things related to the behavioral testing are in need of consideration:

a)Why were mice from 3-6 weeks of age selected for the behavioral tests? 3 weeks just weaned - a time of some serious developmental variations. I'm not asking the authors to test older animals, but a explanation or statement of caveats in the paper would be helpful.

b)Please list odors used in the methods section

c)“we scored for how long mice investigated the odor”. In the methods section please list the operational definition for what you mean by investigating the odor / what was actually assayed.

d)how many total days were animals tested? How many days did they contribute data?

e)were any animals excluded from contributing data beyond the 10 listed in the paper? If so, please state and specify why.

f)“ with free access to food and water throughout the experiment.” is it really true that during the experiment itself there was food and water accessible while the animal was being testing on the 60x60 platform? Perhaps this statement is likely not phrased how intended?

g)perhaps specify that the location of the odor tube and the min oil tube in the two holders was alternated or pseudorandom? If that was indeed the case.

h)it would be helpful if the authors could cite a study which this test was based on to help provide more context to the how's and why's of what they did.

For figure 4 the authors note that, “Note that MC responses to the investigation bouts slowly decrease over time” yet this is not apparent when I look at the simulated traces in Bii.

These statements need supporting citation(s):

“connectivity from AON to ET cells, which have been proposed to be the target of AON projections to the OB glomerular layer.”

“Optogenetically evoked OXT

release increased the duration of social recognition memory, while selective deletion of OXTRs

in the AON decreased memory duration. Interestingly, mice without OXT modulation in the OB

treated social odors in a manner comparable to mice investigating non-social odors, suggesting

that the differences in memory performance when investigating social or non-social odors may

depend on OXT release in response to social odors.”

“These top-down mechanisms were mediated through lowering of the firing threshold in

AON neurons and increased network drive that recruited OB GC interneurons that in turn

provided increased inhibitory output to OB projection neurons MCs.”

“The code/software described in the paper is freely available online at xx.” I am presuming “xx” will be revealed upon publication and is a feature of this double blinded review...and with that we will entrust this inclusion by the production staff and authors.

The first paragraph of the results would be highly suitable in the Introduction.

The second and third paragraphs of the results are a semi-rehashing of the abstract. necessary?

Figure 3 legend: “order responsiveness” should be odor...

Fig 4Aii. The data seem to be means across all 10 mice. Please provide SEMs or StDevs for these data points? Same for Fig 5D please for the data replotted from the Oettl paper (also please make that more clear in the figure legend that these data are from the Oettl paper). Also while at it please correct the alignment/positioning of the x axis numbers which seem to have gotten misplaced somehow.

Fig 4 Bi and Bii - the resolution of these traces is odd and they seem to differ in some ways not expected. Might there be better resolution versions available?

The authors state (in ref to fig 5C) that the responses are significantly higher, though no stats are provided in the results nor in the fig/legend.

There are some minor formatting/syntax issues which would behlepful for the authors to correct prior to the ms being finalized, e.g., in methods: “(McIntyre and Cleland, 2016). .Activity”
